# Plan, recruit, retain: a framework for local healthcare organizations to achieve a stable remote rural workforce

**DOI:** 10.1186/s12960-020-00502-x

**Published:** 2020-09-03

**Authors:** Birgit Abelsen, Roger Strasser, David Heaney, Peter Berggren, Sigurður Sigurðsson, Helen Brandstorp, Jennifer Wakegijig, Niclas Forsling, Penny Moody-Corbett, Gwen Healey Akearok, Anne Mason, Claire Savage, Pam Nicoll

**Affiliations:** 1 The National Centre for Rural Medicine, The Department of Community Medicine, UiT, Tromsø, Norway; 2 Northern Ontario School of Medicine, Lakehead and Laurentian Universities, Thunder Bay and Sudbury, Canada; 3 NHS Highland, Inverness, Scotland; 4 Region Västerbotten, Storuman, Sweden; 5 Akureyri Hospital, Akureyri, Iceland; 6 Qaujigiartiit Health Research Centre, Iqaluit, Canada; 7 NHS Education for Scotland, Centre for Health Science, Inverness, Scotland

**Keywords:** Recruit, Retain, Rural health, Framework, Healthcare personnel

## Abstract

**Background:**

Recruiting and retaining a skilled health workforce is a common challenge for remote and rural communities worldwide, negatively impacting access to services, and in turn peoples’ health. The research literature highlights different factors facilitating or hindering recruitment and retention of healthcare workers to remote and rural areas; however, there are few practical tools to guide local healthcare organizations in their recruitment and retention struggles.

The purpose of this paper is to describe the development process, the contents, and the suggested use of *The Framework for Remote Rural Workforce Stability*. The *Framework* is a strategy designed for rural and remote healthcare organizations to ensure the recruitment and retention of vital healthcare personnel.

**Method:**

The *Framework* is the result of a 7-year, five-country (Sweden, Norway, Canada, Iceland, and Scotland) international collaboration combining literature reviews, practical experience, and national case studies in two different projects.

**Result:**

The *Framework* consists of nine key strategic elements, grouped into three main tasks (plan, recruit, retain). Plan: activities to ensure that the population’s needs are periodically assessed, that the right service model is in place, and that the right recruits are targeted. Recruit: activities to ensure that the right recruits and their families have the information and support needed to relocate and integrate in the local community. Retain: activities to support team cohesion, train current and future professionals for rural and remote health careers, and assure the attractiveness of these careers.

Five conditions for success are recognition of unique issues; targeted investment; a regular cycle of activities involving key agencies; monitoring, evaluating, and adjusting; and active community participation.

**Conclusion:**

The *Framework* can be implemented in any local context as a holistic, integrated set of interventions. It is also possible to implement selected components among the nine strategic elements in order to gain recruitment and/or retention improvements.

## Introduction

Recruiting and retaining a skilled workforce is a common challenge across remote and rural parts of the world. There are different complex and interconnected factors rooted in the wider socio, political, and economic context influencing a health worker’s decision to start, stay, or leave a job in a remote and rural area [1]. The research literature includes a vast pool of studies on different factors facilitating or hindering recruitment and retention of healthcare workers to remote and rural areas. These have been described as “pull” and “push” factors. The “pull” factors are those that attract health professionals for a given job/location. The “push” factors are those that influence the health workers not to take a job or to leave a job in a given location [2].

The three factors most strongly associated with entering a rural job are as follows: (1) a rural upbringing, (2) positive clinical and educational experiences in rural settings as part of undergraduate education, and (3) targeted training for rural practice at the postgraduate level [3]. A range of factors influencing the decision to stay in or leave a rural job have been identified in the literature for a range of health professions in different countries. These factors include financial and economic considerations (such as remuneration and other benefits) [4–7], professional and organizational issues (including professional development opportunities, workload, and infrastructure) [5, 8–13], social factors (including employment and educational opportunities for other family members) [8, 14, 15], individual factors [16, 17], and the characteristics of the local community itself [4, 6, 18–21].

There is a substantial literature proposing and describing interventions taken by different stakeholders to act on this evidence. Interventions can broadly be divided into education policies, monetary incentives, non-monetary incentives, skills substitution, and regulatory policies [1, 2, 4, 22–31]. Although some interventions have shown promise, there is a lack of well-designed studies to measure their short-term and long-term effectiveness in a rigorous fashion [30, 31]. Particularly, there is very little evidence showing the effectiveness of any specific retention intervention [2, 32]. However, support exists in the literature for the effectiveness of “bundling” more than one retention intervention [22, 31, 33].

An additional challenge to successful recruitment and retention is the reality that remote and rural communities all differ from each other [34], even though many of the challenges in rural health are common around the world [35]. The realities of rural settings require healthcare providers to be generalists with a specific broad range of knowledge and clinical skills [36]. The widespread shortages of health professionals mean that gaps may be filled with health professionals who lack the generalist skills and for that reason do not stay. The resulting transience in the workforce adversely affects service quality and patient experience [37].

In this context, local healthcare organizations in remote and rural areas have few practical tools to guide them in their struggle to recruit and retain personnel. Nevertheless, they face this struggle every day. The purpose of this paper is to describe how *The Framework for Remote Rural Workforce Stability* was developed, its contents, and how it can be used. The *Framework* is a strategy designed for rural and remote healthcare organizations to ensure the recruitment and retention of vital healthcare personnel. A strategy is a high-level plan to achieve one or more goals under conditions of uncertainty. Uncertainty refers in this context to the varied and complex research evidence concerning the effects of the different interventions that have been suggested and used in this field of practice, as well as the wider socio, political, and economic context influencing health workers’ job decisions.

## Method—the framework development process

### The partnership

In the period 2011–2019, an international partnership of academics, human resources professionals, health services administrators, health professionals, and social and cultural development professionals, living and working in northern rural or remote communities in Sweden, Norway, Canada, Iceland, and Scotland, has explored factors related to workforce recruitment and retention in rural and remote environments. We synthesized existing research-based knowledge and practical experience to generate new knowledge from case studies in the respective countries. Throughout the period, the goal was to develop a practical tool to guide remote and rural health organizations towards achieving stability in their workforce recruitment and retention activities.

### The setting

The partnership undertook two projects between 2011 and 2019. In the first project entitled Recruit and Retain (2011–2014), funded by EU Northern Periphery Programme 2007–2013, the partnership developed, implemented, and evaluated a variety of initiatives/solutions that were proven to be successful in supporting recruitment and retention in their local communities [38]. This project also developed a composite seven-step business model [39] to assist and underpin the recruitment and retention of healthcare professionals in remote and rural areas.

In the second project Recruit and Retain: Making it Work (2015–2019), funded by EU Interreg Northern Periphery and Arctic Programme 2014–2020, the aim was to utilize the seven-step business model and evaluate its performance. However, it became clear early on that the seven-step model was not sufficiently developed to be useful in practical settings. During the project period, the partnership further refined the seven-step model and developed the broader *Framework* with nine strategic elements and five conditions for success.

### Methodology

The move from the seven-step business model to the *Framework* was based on insights derived from five different case studies, one conducted in each of the partnership countries, and a parallel collaborative Framework development process.

The project was managed collaboratively, with working groups that included representatives from each country. Our initial plan was to create similar local business cases in each partner country as a starting point to try out the seven-step model and design a similar evaluation process to measure and compare outcomes. Early on, it became evident that such a streamlined process was difficult to accomplish in practice. Based on the rural reality, the five case studies eventually dealt with somewhat different issues defined by the local contexts and associated interventions. Some had greater emphasis on planning, while others placed greater emphasis on aspects of recruitment and/or retention. Instead of perceiving this as a problem, we saw this as a stepping-stone for the development of a more real-life-fitted model.

Table 1 gives an overview of the case studies in each country. Specifically, the Swedish case study was focused on recruiting and retaining key personnel to the rural municipality of Storuman [40]; the Norwegian case study aimed at improving the recruitment and stability of regular general practitioners in three rural municipalities [41]; the Canadian case study focused on stabilizing the physician workforce in Nunavut, the most northerly territory of Canada [42]; the Icelandic case study focused on recruiting and retaining specialized physicians in Akureyri Hospital, a rural teaching hospital in the northern part of Iceland [43]; and the Scottish case study was aimed at improving the recruitment and retention of health and social care multi-disciplinary teams in remote and rural Scotland (Highland, Orkney, and Shetland) [44].

**Table 1:** Case studies’ overview: aims and targeted strategic Framework elements

Cases studies		Sweden [40]	Norway [41]	Canada [42]	Iceland [43]	Scotland [44]
Case study aim		*Recruit healthcare personnel to Storuman municipality*	*Improve recruitment and retention of GPs in three case municipalities*	*Stabilize the physician workforce in Nunavut*	*Recruit and retain specialized physicians in Akureyri Hospital*	*Improve recruitment and retention of rural multi-disciplinary teams*
Plan	Assess population service needs		All municipalities evaluated their service model and ended up extending their number of GPs with one extra GP to reduce the workload.			Develop marketing strategies; friendly and informative RR communication processes and information packages; and identify appropriate and accessible education and support.
	Align the service model with population needs			Development of the contract model for new physicians.		
	Develop a profile of target recruits			Inuit/northern physicians serving Inuit.		
Recruit	Emphasize information sharing	Establishing an alumni register to send newsletters with job relevant information to people (approx. 2800) who might be interested in moving back to Storuman.		Development of a cultural orientation app for healthcare providers in Nunavut.	Information meetings with Icelandic medical students in Iceland, Hungary, and Slovakia, and with Icelandic specialists and specialists in training working in Sweden to introduce and promote the hospital.	Accessible user-friendly marketing outlets promoting rural vacancies. Development of an effective template including information on recruit profile, work area, work colleagues, and what rural and remote working in the area is like.
	Community engagement	Establishing a relocation coordination officer in Storuman municipality.			Including a member from the community council in the project group.	Co-designing community information for candidates.
	Supporting spouses/families	Development of a couple recruitment strategy.			Meeting with potential recruits and their families with a member from the municipality to inform of opportunities.	Develop and implement a buddy support system and educational support package.
Retain	Supporting team cohesion					Team approach to developing vacancy adverts.
	Ensure relevant professional development		Establishment of a programme with salaried educational positions for GPs to specialize in family medicine (ALIS-Vest/ALIS-Nord).	Continuing Education and Professional Development (CEPD) events for physicians.	Development of a tailored education programme for new recruits. Some physicians got 3 months extended educational leave to auscultate and do research work.	Piloting of ebook to aid access to evidence based practice. Development of new Multi-Professional Rural Practitioners Programme and Qualification Pathway.
	Training future professionals	Developing a rural education stream as part of the medical school curriculum at Umeå University.		Health careers promotion camp for high school students from around Nunavut.	Work to get accreditation from the Royal College of Physicians to allow Akureyri Hospital to educate specialist in internal medicine and anaesthesia.	Multi-professional partnership package promoting joint training across professions.

A project plan was developed for each case, and project activities were ongoing for 18 months. A case study report template was developed to ensure a common approach to reporting. It included a description of partners and purpose of the collaborative work, project activities and timeline, resources required, narrative descriptions of key outcomes, and lessons learned. All partners took an additional step for creating sustainability plans for the recruitment and retention initiatives addressed in their case study. A common template for this was also developed.

The concrete experiences from the case studies and the long-term perspective built in by the sustainability plans helped to clarify the strategic elements that were eventually incorporated into the *Framework*. The findings from the different case studies were integrated, although not all case studies provided input for every aspect of the *Framework.* A coordinated approach to the wording of the documents and design of the *Framework* was undertaken by a communication working group. The development and fine-tuning of the *Framework* progressed through an iterative process in four in-person workshops where the whole partnership met for several days for updates and discussions, and by virtual steering group meetings held on a regular basis throughout the project period.

### Reflexivity

The *Framework* was developed through a protracted reflexive process in which the topic of recruitment and retention was investigated from many different starting points and approaches. The long duration of this collaborative work provided all partners with time to reflect on and validate the relevance of the different aspects of the *Framework*. Common elements of workforce recruitment and retention that are possible to address regardless of local context were identified even though each partner worked within different settings and health systems. Validation occurred through the process of testing concepts against the literature, including the previous Recruit and Retain project, and practical experiences in the five case studies. In addition to the four face-to-face workshops, the prototype *Framework* was presented also at conferences so that interested colleagues beyond the project partners contributed to the validation process.

## Results—The Framework for Remote Rural Workforce Stability

The *Framework* consists of nine key strategic elements, grouped into three main tasks (plan, recruit, retain). To be an effective strategic tool, five conditions for success should be satisfied. The *Framework* describes the necessary elements of an overall strategy to ensure the recruitment and retention of the right professionals to provide needed services in rural and remote locations.

### Underpinnings

Many remote rural organizations are caught in a continuous cycle of recruiting to fill vacancies and often appoint service providers who are not well prepared for the service requirements or the community context. Service quality and patient experience are adversely affected when their service providers are largely transient. A long-range strategy that ensures workforce sustainability should include three levels of priorities:

1. Make inter-sectoral investment in training and career promotion. If possible, recruit people from the local community or region and develop cultural relevance of the services provided. This will increase the likelihood that the professionals will stay.
2. Create a desirable workplace. This includes a cohesive team and supportive management, a safe and well-equipped clinical work environment, and broadband internet. Emphasis should be on recruiting and retaining people who will make the remote rural community their home.
3. Create and incentivize a pool of transient workers who make a longer-term commitment to the remote rural region. There will always be a need for temporary workers to fill vacations, maternity leaves, and other temporary vacancies. It is possible to build a pool of repeat candidates who contribute to the continuity and quality of service in the region.

### Five conditions for success

We have identified that pan-organizational buy-ins formed by committed top-level leadership in local, regional, and national governments who advocate for and support initiatives to recruit and retain vital workforce are important requirements for the *Framework* to be effective. The additional following conditions are essential to the successful implementation of the *Framework*:

1. Recognition of unique rural and remote issues. Life and work in rural and remote locations are unique and different from urban settings. Policy and programme decisions must take this into account. Remote communities are also generally distinct from one another, and interventions need to be tailored to specific communities if they are to have the desired impact.
2. Active community participation or engagement is an important element of the *Framework* and should be a part of regional and national planning for rural and northern workforce initiatives so that rural and remote perspectives are reflected in policies and programmes. The vision must be “nothing about us, without us”.
3. Targeted investments and dedicated resources must be provided. Success is most likely when the investments are additional to rather than within existing budgets.
4. An annual cycle of key recruitment and retention activities must be identified and undertaken. Building these activities into job descriptions and performance standards ensures that initiatives are future-focused and receive attention.
5. The work must be monitored, evaluated, and modified on a continuous basis, with a strong emphasis on learning from practical experiences and continuous quality improvement.

### Nine key strategic elements

**Fig. 1:**
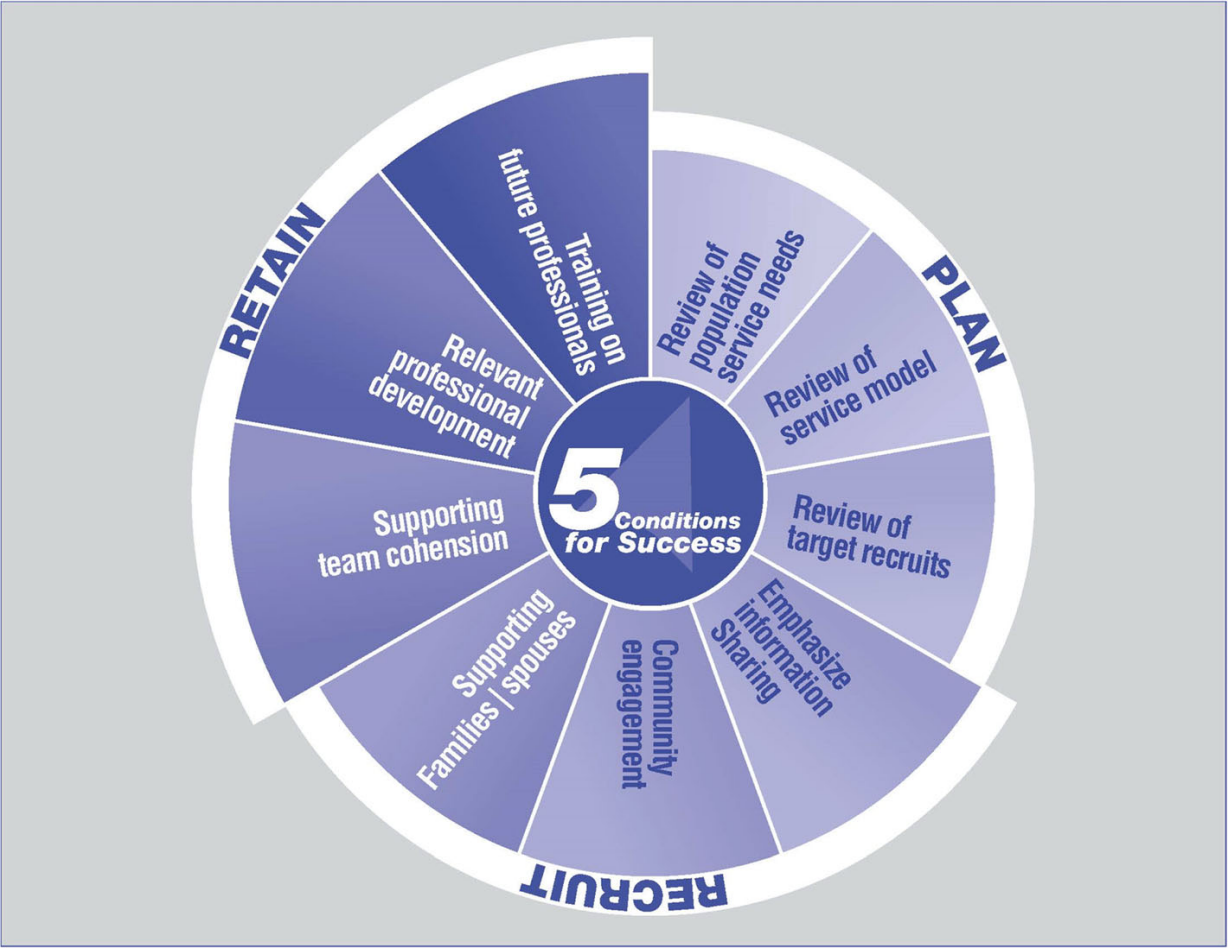
The Framework for Remote Rural Workforce Stability

Figure 1 illustrates how the strategic elements of the *Framework* are grouped into three main tasks: plan, recruit, and retain. The elements are placed around a circle to illustrate that there is no definite starting or endpoint.

#### Plan

These three elements are activities that may be undertaken at local, regional, and national levels.

##### Assess population service needs

A socially accountable organization designs its services to meet the needs of the population it serves [45]. This implies having systems in place to regularly assess the population’s (changing) needs. Needs assessments typically include analyses of the population’s demographics, the burden of acute and chronic disease, waiting times for various services, and distances to specialized services. It is strongly recommended that an evidence-informed approach be taken to develop data sources that accurately assess service needs for the targeted population and that a plan is implemented to routinely monitor any changes to the population’s specific needs.

##### Align the service model with population needs

Successful health-service models are explicitly “contextualized” to the local environment, developed in the community, by the community, for the community. Rural health services are often modelled on urban services which may be counterproductive and threaten workforce stability. When service needs cannot be met by professionals in the existing service model, burnout and job dissatisfaction for even the most committed professionals can be the result. It is a misuse of resources to try recruiting and retaining healthcare personnel into a poorly designed and outdated service model.

##### Develop a profile of target recruits

In rural and remote environments, management and their human resource teams may be obliged to hire whoever is readily available and ultimately be disappointed with the outcome. Delivery of safe and effective healthcare in remote and rural areas requires a specific additional skillset. When compared to their metropolitan counterparts, rural practitioners are “extended generalists”. Rural practitioners provide a wider range of services and carry a higher level of clinical responsibility in relative professional isolation [36]. They require ongoing skills maintenance and continuing education. Organizations are encouraged to seriously consider the characteristics of the person they would like to hire and then target promotion and advertising materials to this profile.

#### Recruit

These elements are generally led at the local and/or agency level.

##### Emphasize information sharing

Making a move to a rural or remote community, relocating and living there is a major consideration. Prospective employers should seek to reach recruits with more than just a job advertisement. Prospective recruits require accessible comprehensive information that is likely to influence them in making this major life decision. Professionals may have families including a spouse who needs to find work, and children requiring education and social and recreational activities. Making it easy for recruits to access information about a community through online resources and dedicated personnel answering emails and calls for information may help families choose one rural location over another. Providing opportunities for potential recruits to have personal and positive contact with recruiters, with current employees, and with community members is an important part of the recruitment process.

##### Community engagement

Active involvement of communities in defining their recruitment and retention strategy is essential to the development of partnerships that will ensure that the entire suite of interventions works. Having communities involved in defining the approach that will be used in their community ensures that solutions are feasible in their specific environment and that community members are more likely to sustain them. Involving communities in the planning and development of their own healthcare and other essential services encourages customized processes using local knowledge and addressing local concerns.

##### Supporting spouses/families

Ensuring that the employee and family are made to feel welcome in the community and supported to become integrated in community social, recreational, and other activities is a key factor in ensuring a positive start and long stay in the community. This can mean proper housing, involving community partners in meeting with the new recruits and their families, giving tours of the town, health services, and schools to ensure they are able to register in recreational and other programmes. Lack of work opportunities for spouses is known to be a key barrier in the recruitment of professionals to rural locations. It is often one of the most challenging factors to mitigate. Dedicating resources to assisting spouses to learn about work opportunities is a good start to addressing this barrier. Partnering with other employers to secure employment for spouses is more challenging, however, likely to have a significant impact on recruitment.

#### Retain

These elements are activities that may be undertaken at a local, regional, and/or national level.

##### Supporting team cohesion

In rural and remote communities, professionals often work in isolation, without immediate access to specialist support that they may have enjoyed in previous urban roles or during their training. In a service environment, often with high demands, and limited resources, professionals can feel stretched thin, unsupported, and frustrated at their inability to make system changes. Rural and remote health leaders who have overcome challenges in recruitment and retention of professionals typically report that they consider supporting team cohesion to be a major part of their role. They involve their team of professionals in decisions on who to recruit to the team; they create opportunities for their team to socialize and learn together, and offer them some control over their work environments (shift scheduling, joint posts, rotational posts, strategic planning, creation of leadership roles among professionals, such as regional professional development lead). Leadership skills involve inspiring trust and respect, as well as motivating action among team and community members.

##### Ensure relevant professional development

Professionals working to deliver safe and effective healthcare within remote and rural communities require a broad range of skills supported by ongoing access to education, training, and skills maintenance that are relevant to their practice context. Consequently, high-quality professional development is a key contributor to successful retention including local professional development involving the health team, adoption of cascading training models, online professional development, and funded travel for specific professional development programmes, skills developments, and updates. Unfortunately, rural practitioners often travel to urban centres and undertake training that lacks relevance to their rural practice and context within which they provide care.

##### Training future professionals

Developing an academic/training mandate for an organization and seeking funds to allow healthcare teams to dedicate time to training the professionals of the future will lead to a strong return on investment. There is a clear and substantial body of evidence which confirms that offering health professionals training in rural and remote environments leads to greater retention of those professionals. Furthermore, training in rural and remote environments ensures that professionals have the broad range of skills that are needed for rural practice.

Rural communities can strive to become centres of rural training excellence, contributing to a strong rural training programme for all remote and rural staff, or they may wish simply to take the necessary steps to receive students on rural placements a few times per year. Any effort on this spectrum is likely to have multiple positive impacts on recruitment and retention.

Table 1 provides information about the presence of the different key strategic elements in the five case studies. The table illustrates the flexibility of the Framework and the possibility to concentrate on selected elements among the total nine, but it is important to keep in mind that the case studies helped develop the Framework—not to test it out.

## Discussion

The *Framework for Remote Rural Workforce Stability* identifies actions that can be taken by various levels of government and by local agencies. Local or regional agencies can use this *Framework* to initiate dialogue with federal governments about their shared role in advancing rural and remote health services. The goal in any community or region would be to identify which elements of the *Framework* are likely to have the greatest impact in their local reality, then design a set of interventions to implement them and move towards long-term workforce stability [32].

The *Framework* can be implemented as a holistic, integrated set of interventions. However, it is not a recipe to be followed precisely or in any particular sequence, to achieve results. The available human resources and time might be limited in real-life settings. It was, therefore, important to develop a flexible tool from which it is possible to implement selected strategic elements among the total nine. Based on our case studies, we know that concentrating effort into one or a few of the nine strategic elements can give recruitment and/or retention improvements.

Our research and development project was limited in its ability to achieve the original goal of a consistent framework for implementation and evaluation across countries. Although rural and remote communities have much in common with each other across jurisdictional boundaries [35], it was clear early in the project that each country participating in this research study was in a unique state. As a result, the *Framework*, a robust, evidence-informed toolkit, was developed and is now ready for implementation and further validation in different countries around the world. There is substantial potential for further research and for practical experience in utilizing the *Framework.* Further research into implementing the *Framework* will need to be informed by changing expectations among health professionals who are potential recruits into remote rural health services [46].

Our experience was that rural communities often have more in common with rural communities in other countries than they do with urban centres within their own national borders. Investing in training of people from rural and remote communities, in rural and remote locations, for rural and remote jobs, leads to more successful recruitment and stability of services in these locations [3, 36]. However, every remote rural community is unique. Active community participation is essential to ensure the success of initiatives that target remote rural communities [47, 48]. Top-down initiatives are doomed to fail.

As pointed to in the “Introduction” section, there is substantial literature describing individual interventions by different stakeholders that are more or less effective in recruiting and retaining healthcare personnel in rural and remote areas [1, 2, 22, 32]. In contrast, the literature presents very few practical and actionable tools to undertaking this complex and multi-faceted task. Cosgrave [49] points to the fact that most existing frameworks tend to be highly complex, including comprehensive lists of factors involved in recruitment and retention. While such models likely assist in building understanding around the complexity of the rural health workforce issue, they do not necessarily support the development of strategic, practical actions. The problem is now well understood, and the impacting factors clearly identified [22, 50]. However, despite this strong evidence base, rural health services and their communities remain unclear about the actions they can or should take to improve their recruitment and retention situation. The utilization and further validation of the *Framework* will be an indication of how suitable it is in tackling real-world recruitment and retention problems in remote and rural areas. We welcome additional research on recruitment, retention, and workforce stability outcomes from the use of the *Framework* in different local contexts around the world.

There are already examples of implementing the *Framework* in other settings. In January 2019, the *Framework* was launched via a multisite video forum at which the five country case studies were presented, and each partner began the process of exploring the potential for implementing the *Framework* in different settings. In Canada, the *Framework* provided the basis for workshop discussions focused on the *Physician Resources Action Plan for Northern Ontario* that had been developed following “Summit North: Building a Flourishing Physician Workforce in Northern Ontario” in January 2018 [51]. Specifically, there was exploration of community engagement: whom to engage, what to discuss, and how to engage. Small group discussions then fleshed out: the conditions for success drawing on the partnership pentagram (policymakers, health service administrators, healthcare providers, academic institutions, and communities) [52], leadership commitment, and monitoring and evaluation.

The Norwegian Ministry of Health and Care Services and the Colombian Ministry of Health and Social Protection have signed a Memorandum of Understanding on health cooperation called *Rural Health for Peace*. Among the issues to be developed are primary healthcare and health services in rural and remote areas. The *Framework* is being used as the basis for collaborating with local small communities, health agencies, and academic institutions to enhance the quality, effectiveness, and sustainability of healthcare in Tolima province. The *Framework* has been translated into Spanish and adapted to the Colombian context and is guiding specific research and development initiatives. Community engagement is a key feature of *Rural Health for Peace*, actively involving local communities including former FARC—Revolutionary Armed Forces of Colombia (People’s Army) combatants.

In Scotland, the *Framework* has been included as a key element within a proposal to develop a *Centre of Excellence (CoE) for Remote, Rural and Island Healthcare* aimed to improve and innovate health and care provision including the recruitment, retention, and support for health and care staff. The CoE proposal has been co-produced by a multi-agency and community working group led by NHS Education for Scotland and has been submitted to the Scottish Government in response to recommendations made within the Sir Lewis Ritchie Report [53].

In Sweden, the *Framework* functions as a backbone for the local healthcare district of South Lapland-Region Västerbotten’s transformation of primary healthcare services, as one of four model areas in Sweden connected to the Swedish primary care reform. It is also used to structure a study through the Nordic Council of Ministers, which aim to give voices for how digital transformation of healthcare and social care services can influence recruitment and retention possibilities.

The preliminary work for Recruit and Retain: Making it Work focused on health services with an additional strategic focus on the broader public sector, and across the international collaborative. In the next phase, many partners extended their case studies beyond health services to education and other essential public services. In addition, engagement with the private sector operating in rural and remote environments including mining, retail, and regional economic development organizations confirmed that the rural private sector faces similar personnel recruitment and retention challenges and can benefit from applying this *Framework*.

## Conclusion

The *Framework for Remote Rural Workforce Stability* is a result of transnational collaboration and a practical everyday tool that can be implemented in any local context as a holistic, integrated set of interventions or as selected components to gain recruitment and/or retention improvements. Moreover, it can promote dialogue across jurisdictional lines about how to address the high cost and limited effectiveness of services in rural and remote areas that struggle with workforce instability.

## Data Availability

Not applicable.
